# Clinical course of five patients definitively diagnosed with idiopathic perilymphatic fistula treated with transcanal endoscopic ear surgery

**DOI:** 10.3389/fneur.2024.1376949

**Published:** 2024-03-15

**Authors:** Toshinori Kubota, Tsukasa Ito, Takatoshi Furukawa, Hirooki Matsui, Takanari Goto, Chikako Shinkawa, Han Matsuda, Tetsuo Ikezono, Seiji Kakehata

**Affiliations:** ^1^Department of Otolaryngology, Head and Neck Surgery, Yamagata University Faculty of Medicine, Yamagata, Yamagata, Japan; ^2^Department of Otolaryngology, Yonoezawa City Hospital, Yonezawa, Yamagata, Japan; ^3^Department of Otolaryngology, Yamagata Prefectural Shinjyo Hospital, Shinjo, Yamagata, Japan; ^4^Department of Otolaryngology, Saitama Medical University, Iruma-gun, Saitama, Japan

**Keywords:** idiopathic perilymphatic fistula, cochlin-tomoprotein, transcanal endoscopic ear surgery, sudden sensorineural hearing loss, disequilibrium

## Abstract

**Objectives:**

An idiopathic perilymphatic fistula (PLF) can be difficult to diagnose because patients present with sudden sensorineural hearing loss (SSHL) and/or vestibular symptoms without any preceding events. In such cases, we currently test for cochlin-tomoprotein (CTP) to confirm the diagnosis of idiopathic PLF because CTP is only detected in the perilymph. In this study, we report the clinical course of five patients definitively diagnosed with idiopathic PLF who underwent PLF repair surgery using transcanal endoscopic ear surgery (TEES).

**Patients and methods:**

Five patients were initially treated with intratympanic dexamethasone for SSHL, at which time a CTP test was also performed (preoperative CTP test). Due to refractory hearing loss and/or fluctuating disequilibrium, PLF repair surgery using TEES was performed to seal the oval and round windows using connective tissue and fibrin glue. These patients were diagnosed with definite idiopathic PLF based on pre- or intra-operative CTP test results (negative, < 0.4 ng/mL; intermediate, 0.4–< 0.8 ng/mL; and positive, > 0.8 ng/mL). We evaluated pre- and intra-operative CTP values, intraoperative surgical findings via a magnified endoscopic view, and pre- and post-operative changes in averaged hearing level and vestibular symptoms.

**Results:**

Pre- and intra-operative CTP values were positive and intermediate in three patients, positive and negative in one patient, and negative and positive in one patient. None of the patients had intraoperative findings consistent with a fistula between the inner and middle ears or leakage of perilymph. Only two patients showed a slight postoperative recovery in hearing. Four patients complained of disequilibrium preoperatively, of whom two had resolution of disequilibrium postoperatively.

**Conclusion:**

A positive CTP test confirms PLF in patients without obvious intraoperative findings. The CTP test is considered more sensitive than endoscopic fistula confirmation. We consider that CTP test results are important indicators to decide the surgical indication for idiopathic PLF repair surgery. In our experience with the five cases, two of them showed improvements in both hearing and vestibular symptoms.

## 1 Introduction

A perilymphatic fistula (PLF) is defined as an abnormal communication between the perilymph-filled inner ear and the air-filled space of the middle ear. A PLF can be caused by internal or external trauma, but it can also be idiopathic. The diagnostic criteria for PLF in Japan were revised by the Intractable Hearing Loss Research Committee of the Ministry of Health, Labor, and Welfare in 2016. The PLF criteria in Japan acknowledge that idiopathic cases exist.

Idiopathic PLF can be difficult to diagnose because patients present with sudden sensorineural hearing loss (SSHL) and/or vestibular symptoms without any abnormalities demonstrated on an MRI or CT scan. In such cases, we currently test for cochlin-tomoprotein (CTP) in the middle ear to confirm idiopathic PLF because CTP is detected only in perilymph (1, 2). Suspected idiopathic PLF has been surgically treated via microscopic ear surgery (MES), but MES is not guaranteed to resolve SSHL and vestibular symptoms. Transcanal endoscopic ear surgery (TEES), however, offers a less invasive option for such patients, and TEES may have the potential to visualize fistulas/microfissures more clearly than MES. In this study, we report the clinical course of five patients definitively diagnosed with idiopathic PLF who were treated with PLF repair surgery using the TEES technique.

## 2 Patients and methods

### 2.1 Patients

The five patients (three male individuals and two female individuals; age range, 15–83 years; median age, 52.0 years) we present were initially treated with intratympanic dexamethasone (IT-DEX) for SSHL at Yamagata University Hospital between 2014 and 2017. IT-DEX was performed in the hospital for 8 consecutive days (3). Hearing levels were not completely recovered in all cases. These patients had refractory hearing loss and/or fluctuating disequilibrium; idiopathic PLF was considered based on the CTP test results at the time of IT-DEX and the course of hearing levels and/or vestibular symptoms. After informed consent, we performed a PLF repair surgery using the TEES technique to seal the inner ear windows with the hope of improving hearing and vestibular symptoms.

### 2.2 Diagnostic criteria for PLF

The Japanese PLF diagnostic criteria, which were revised by the Intractable Hearing Loss Research Committee of the Ministry of Health, Labor, and Welfare in 2016, were used to diagnose PLF in the current study (Table 1) (4). These criteria for PLF show that PLF is definitively diagnosed based on symptoms and positive laboratory findings. PLF symptoms include hearing loss, tinnitus, and vestibular symptoms. There are two laboratory findings of significance in patients with PLF: visual identification of a fistula between the middle and inner ears by microscope or endoscope; and a biochemical test that detects perilymph-specific proteins, including CTP, from the middle ear.

**Table 1:** Diagnostic criteria in Japan for PLF.

A. Symptoms Hearing impairment, tinnitus, aural fullness, and vestibular symptoms are observed in cases who had preceding events, as listed below: Coexisting or pre-existing middle and/or inner ear diseases (trauma, cholesteatoma, tumor, anomaly, SCCD, etc.) middle and/or inner ear surgeries Barotrauma caused by antecedent events or external origin (e.g., blasting, diving, or flying) Barotrauma caused by antecedent events of internal origin (e.g., nose-blowing, sneezing, straining, or carrying heavy objects) B. Laboratory findings (1) Microscopic/endoscopic inspection Visual identification of fistula(s) between the middle and inner ear by a microscope or endoscope. Fistulas can develop at the cochlear window, vestibular window, fracture site, microfissure, malformation, destruction in the bony labyrinth caused by inflammation, etc. (2) Biochemical test Perilymph-specific protein is detected in the middle ear C. Reference A perilymph-specific protein, e.g., Cochlin-tomoprotein (CTP) detection test. After myringotomy, the middle ear is rinsed with 0.3 mL of saline three times, the fluid recovered (middle ear lavage (MEL)) and tested by polyclonal antibody ELISA. The cutoff criteria are: 0.4 < CTP negative; 0.4 ≤ CTP <0.8 intermediate; 0.8 ≤ CTP positive Idiopathic cases may exist The following symptoms and/or test results may be observed: Streaming water-like tinnitus or feeling of running water in the middle ear Popping sound can be heard at the onset Nystagmus and/or vertigo induced by pressure application to the middle ear (Hennebert’s phenomenon, fistula sign) Imaging studies may show a fistula in the bony labyrinth or pneumolabyrinth Progression of hearing impairment, tinnitus, aural fullness may be acute, progressive, fluctuating, or recurrent The main complaints can be vestibular symptoms without hearing impairment D. Differential diagnosis Inner ear diseases have known causes, such as viral infection, genetics, and vestibular schwannoma. E. Diagnosis Probable PLF: Only symptoms listed in A Definite PLF: Symptoms and laboratory findings listed in B

### 2.3 CTP test

Ikezono et al. (1) identified CTP as a perilymph-specific protein. Therefore, the detection of CTP in the middle ear is consistent with a PLF with perilymph leakage. CTP was quantified using polyclonal antibody ELISA with a specimen obtained from the middle ear cavity. The middle ear cavity was rinsed three times repeatedly with 0.3 mL of saline, and then saline was collected as a specimen for the CTP test. The cutoff criteria tested by polyclonal antibody ELISA are shown in Table 1 (negative, < 0.4 ng/mL; intermediate, 0.4–< 0.8 ng/mL; and positive, > 0.8 ng/mL). The sensitivity and specificity of the CTP test by polyclonal antibody ELISA are 86.4 and 100%, respectively (2).

All the samples were measured by T.I. in a blinded fashion, using a quality-controlled, standardized methodology established in collaboration with the central pathology lab (SRL Inc.).

The CTP test using polyclonal antibody ELISA was a prospective clinical study that was conducted with the approval of the Ethics Committee of Yamagata University (H28-303) and Saitama Medical University Hospital (IRB No. 13086). Informed consent was given by the patients. CTP tests were performed twice, as follows: the first during the IT-DEX procedure (preoperative CTP test) and the second during the TEES procedure (intraoperative CTP test).

The CTP test was performed in all patients who underwent IT-DEX for SSHL and provided informed consent for the CTP test. The CTP-positive rate for SSHL in our institutions during the study period was 15.1% (8/53). PLF repair surgery was performed in four of eight CTP-positive patients.

### 2.4 PLF repair surgery using the TEES technique

Our indications for idiopathic PLF repair surgery were as follows: Surgical indication 1: Patients with symptoms and/or test results suggestive of PLF, such as progressive hearing loss [Table 1 (4)]. Surgical indication 2: Patients with a positive CTP test whose hearing has not completely recovered. Surgical indication 3: Patients with positive CTP results and recurring hearing loss and/or vestibular symptoms.

PLF repair surgery using the TEES technique was performed under general anesthesia. Angled rigid endoscopes (0 and 30 degrees) with an outer diameter of 2.7 mm (KARL STORZ, Tuttlingen, Germany) were used. Images were recorded through a full high-definition (HD) camera (KARL STORZ), which was attached to the endoscope lens, and these images were displayed on a full HD monitor.

**Figure 1:**
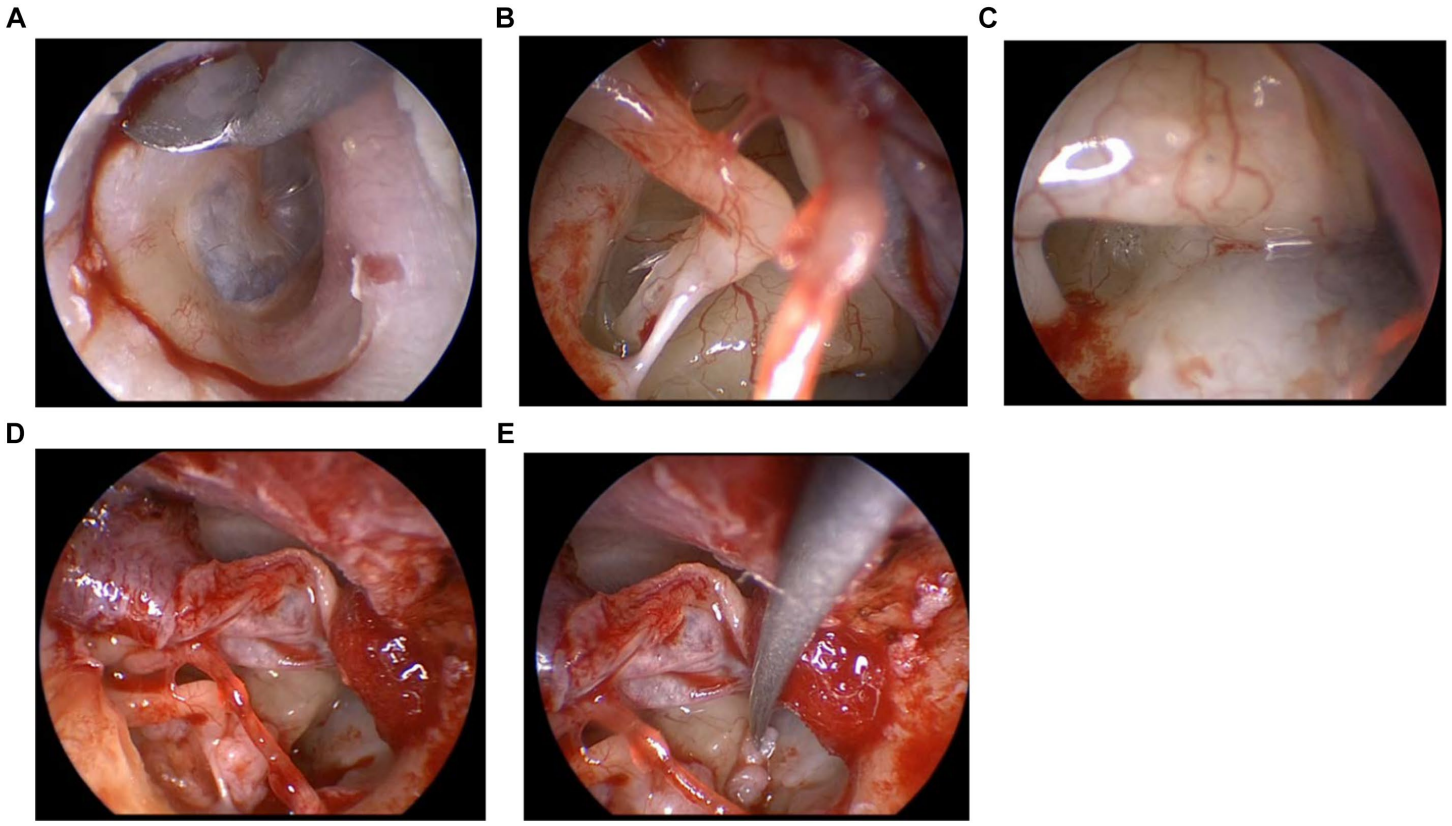
**(A)** Tympanomeatal flap was elevated. **(B)** Observation of the oval window. **(C)** Observation of the round window. **(D)** Sealing the area surrounding the stapes with connective tissue. **(E)** Sealing the round window niche with connective tissue.

In the patient with right ear disease, a tympanomeatal flap was elevated with a circumferential incision from the 6 o’clock to the 12 o’clock position (Figure 1A). After elevation of the tympanomeatal flap, the middle ear was checked for the presence or absence of perilymphatic leakage for approximately 10 min (Figures 1B,C). The middle ear cavity was rinsed three times with 0.3 mL of saline for the CTP detection test. A minimum transcanal atticotomy was performed to clear the surgical field around the oval window. The mucosa around the oval and round windows was scratched for the attachment of the connective tissue graft. The area surrounding the stapes and round window niche was sealed with connective tissue obtained from the postauricular subcutaneous area (Figures 1D,E). Finally, fibrin glue was used to fix the connective tissue. The tympanomeatal flap was put back, and the external ear canal was then packed with chitin sheets, Gelfoam sponges soaked in antibiotic eardrops, and Merocel sponges.

### 2.5 Assessment of hearing levels and vestibular symptoms

Hearing tests and vestibular symptom assessments were performed preoperatively and postoperatively. Changes in hearing levels in each patient were assessed using the criteria established by the Acute Severe Hearing Loss Study Group, which are as follows: “Complete recovery” signifies recovery to a hearing level within 20 dB in pure tone audiometry (PTA) or to the same hearing level as the unaffected side; “Marked recovery” refers to more than 30 dB of recovery in PTA; “Slight recovery” is a recovery of 10–29 dB; and “No response” corresponds to a recovery of less than 10 dB (5). Changes in vestibular symptoms were assessed on the basis of the patient’s subjective complaints, and the results were categorized as unchanged, progressing, or demonstrating marked improvement. Patient data were thoroughly analyzed and followed up for more than 6 months postoperatively (range, 0.7–4.2 years; median, 2.1 years).

## 3 Results

Table 2 shows the clinical courses for the five patients. The indication for surgery in each case was determined from the following events: Patient #1 had progressive hearing loss. Patient #2 had a positive CTP result and no recovery in hearing. Patient #3 showed marked recovery in hearing after IT-DEX, but he had recurrent disequilibrium. Patient #4 showed no recovery in hearing after IT-DEX, and he had recurrent disequilibrium. Patient #5 showed hearing improvement to 43 dB after IT-DEX but then experienced repeated hearing loss and disequilibrium (Table 3).

**Table 2:** Clinical courses of five patients.

No.	Age	Gender	Symptoms	Time to IT-DEX (day)	Time to surgery (day)	Preoperative CTP test (ng/ml)	Intraoperative CTP test (ng/ml)	Fistula finding at surgery	Hearing levels Pre IT-DEX	Hearing levels Preoperative	Hearing assessment Post IT-DEX	Hearing levels Postoperative	Hearing assessment Postoperative	Improvement of vestibular symptoms
1.	15	Female	Hearing loss, disequilibrium	6	19	<0.20	1.13	None	89 dB	111 dB	No response	99 dB	Slight recovery	Demonstrating marked improvement
2.	43	Male	Hearing loss	12	58	0.81	0.48	None	77 dB	77 dB	No response	84 dB	No response	-
3.	52	Male	Hearing loss, disequilibrium	8	192	3.44	<0.20	None	83 dB	37 dB	Marked recovery	38 dB	No response	unchanged
4.	81	Male	Hearing loss, disequilibrium	35	416	1.85	0.53	None	48 dB	51 dB	No response	47 dB	No response	unchanged
5.	83	Female	Hearing loss, disequilibrium	3	727	1.00	0.48	None	57 dB	69 dB	Slight recovery*	43 dB	Slight recovery	Demonstrating marked improvement

^*^Patient #5 showed hearing improvement to 33 dB after IT-DEX, but then experienced repeated hearing loss and disequilibrium. She had no hearing loss or disequilibrium attacks for 2.5 years after surgery.

**Table 3:** Surgical indications for idiopathic PLF repair surgery in our institution.

Surgical indication 1: Patients with symptoms and/or test results suggestive of PLF, such as progressive hearing loss. (Patient #1) Surgical indication 2: Patients with positive CTP results and hearing not completely recovered. (Patient #2) Surgical indication 3: Patients with positive CTP results and recurrent hearing loss and/or vestibular symptoms. (Patients #3, #4, and #5)

The association between pre- and intra-operative CTP values was positive and negative in 1 patient (patient #3), positive and intermediate in 3 patients (#2, #4, and #5), and negative and positive in 1 patient (#1). No fistulas were identified during endoscopic surgery in any patients. Two patients showed a postoperative slight recovery in hearing (#1, #4.). Four patients complained of disequilibrium preoperatively, of whom two had demonstrated marked improvement of disequilibrium postoperatively (patients #1 and #5).

## 4 Discussion

PLFs are classified into four categories according to etiology in Japan (Table 4) (4). A Japanese multicenter study involving 497 cases with suspected PLFs reported that 125 cases were classified as category 1, 28 as category 2, 77 as category 3, and 192 as category 4. The CTP-positive rate has been reported to be 8–50% for category 1 (depending on the type of disorder), 14% for category 2, 23% for category 3, and 19% for category 4 (4). Sasaki et al. reported that the CTP-positive rate was 20% in 56 SSHL patients with no apparent antecedent event and significantly higher CTP values in those aged 60 years and older (6). The presence of idiopathic PLF pathology corresponding to category 4 was considered to be present, as evidenced by the recognition of a case in category 4 in which CTP positivity led to a definitive diagnosis of PLF.

**Table 4:** Categorization of PLF used in Japan.

Category 1: linked to trauma, middle and inner ear diseases, middle, and/or inner ear surgeries Category 2: linked to barotrauma caused by antecedent events of external origin (such as flying or diving) Category 3: linked to barotrauma caused by antecedent events of internal origin (such as straining, sneezing, or coughing) Category 4: has no apparent antecedent event

The five patients evaluated in this study underwent CTP testing twice (once at the time of IT-DEX [preoperative CTP test] and once at the time of PLF repair surgery using TEES [intraoperative CTP test]). Changes in the status of PLF can be inferred from changes in pre- and intra-operative CTP values. A patient who is preoperatively CTP-positive and intraoperatively CTP-intermediate indicates the possibility of persistent or recurrent PLF. We assumed that perilymphatic leakage and spontaneous closure of the PLF were repeated in patient #5, thus the hearing and disequilibrium improved after surgery, even after 727 days from the onset of the disease to the surgical treatment. A patient who was preoperatively CTP-positive and intraoperatively CTP-negative suggested the possibility of spontaneous PLF closure. A patient who was preoperatively CTP-negative and intraoperatively CTP-positive indicated the possibility that the PLF closed spontaneously and reoccurred or that the preoperative CTP test was a false-negative. It is important to note that a CTP-negative does not rule out the PLF, because the condition of the PLF can change during the clinical course of the patient.

Among the five patients with idiopathic PLF in this study, none of the patients achieved complete hearing recovery after IT-DEX treatment. Sasaki et al. (6) reported that CTP-positive patients with SSHL had a significantly worse hearing recovery rate after IT-DEX treatment. Although steroids may be more easily transmitted to the inner ear in PLF conditions, the results of the study suggest that steroids may be less effective in the treatment of idiopathic PLF. The therapeutic efficacy of steroids in idiopathic PLF is unclear.

We have been using TEES for ear surgery since September 2011. In recent years, TEES has gained popularity and is being applied to different types of middle ear diseases, including PLF, as a result of advances in imaging technology, including full HD video systems. TEES using a full HD video system offers a number of advantages over MES, such as a wide field of view, higher magnification of fine anatomic structures, and clear visualization of anatomic areas that are located in blind spots when viewed through a microscope (7–9). Matuda et al. reported a category 2 PLF case with a congenital dehiscence of the stapes footplate (10). In contrast, the patients in the present study were definitively diagnosed with PLF based on the CTP test results, but a fistula could not be confirmed, even via a magnified endoscopic view. In the diagnosis of idiopathic PLF, the CTP test is considered more sensitive than endoscopic fistula confirmation.

Surgical treatment of idiopathic PLF (in which no fistula can be identified) involves sealing the round and oval windows using connective tissue, temporal fascia, or tragal perichondrium (11). Based on our experiences, PLF repair surgery using TEES for idiopathic PLF can be performed without complications under clear visualization and a wide field of view. Heilen et al. (12) reported that there is no significant hearing improvement after exploratory tympanotomy with sealing of the round window for patients with SSHL. Prenzler et al. (13) reported that exploratory tympanotomy with sealing of the round and oval windows is useful in patients with profound SSHL. Matsuda et al. stated that indications for idiopathic PLF repair surgery are patients whose chief complaint is sustained vestibular symptoms, and the onset or exacerbation of vestibular symptoms was accompanied by sudden, fluctuating, and progressive hearing loss. They reported that the surgery was performed in seven patients, with five showing improved vertigo within 1 week and one within 1 month (14). In our experience with TEES repair surgery, two of five patients showed improvements in both hearing and vestibular symptoms, but patient #2 showed no response in hearing, and 2 patients (#3 and #4) showed unchanged vestibular symptoms. Taking into account these reports and the results of the current study, it is unclear whether sealing inner ear windows for idiopathic PLF improves hearing loss or vestibular symptoms. Large multicenter studies are needed to determine the treatment efficacy of PLF repair surgery via TEES or MES for idiopathic PLF.

The timing of repair surgery is another treatment issue for PLF patients. The need for early PLF repair surgery is unclear because some PLFs may close spontaneously. Matsuda et al. (14) reported that the time to surgery was significantly shorter in cases with good hearing recovery of more than 10 dB postoperatively compared to those with no improvement. We should consider early PLF repair surgery as a treatment option for CTP-positive patients whose hearing and vertigo do not improve after systemic steroid administration or IT-DEX. The efficacy of early PLF repair surgery in patients with idiopathic PLF should be evaluated in further studies.

The CTP test is a sensitive and useful examination in the diagnosis of idiopathic PLF and in determining the indications for PLF repair surgery, but it has a major shortcoming; specifically, it takes approximately 1 month to obtain test results. Thus, early diagnosis and early surgical treatment are difficult in patients with idiopathic PLFs because they have no apparent antecedent events associated with PLFs. The development of rapid testing methods for CTP is desirable in the future. If early diagnosis of idiopathic PLF becomes possible, surgical treatment can be performed earlier and surgical outcomes can be improved. Another limitation of the CTP test is that a positive CTP result may be derived from residual CTP in the middle ear cavity even after the PLF has been closed, but the development of rapid tests for CTP may reduce this limitation.

## 5 Conclusion

We evaluated five patients definitively diagnosed with idiopathic PLF who underwent PLF repair surgery using TEES. PLF repair surgery using TEES can visualize potential fistulas/microfissures more clearly than MES; however, we have not identified any in these five cases. The CTP test is considered more sensitive than endoscopic fistula confirmation. We consider that CTP test results are important indicators to decide the surgical indication for idiopathic PLF repair surgery. In our experience with five cases, two of them showed improvements in both hearing and vestibular symptoms.

## Data availability statement

The raw data supporting the conclusions of this article will be made available by the authors, without undue reservation.

## Ethics statement

The studies involving humans were approved by the Ethics Committee of Yamagata University and Saitama Medical University Hospital. The studies were conducted in accordance with the local legislation and institutional requirements. Written informed consent for participation in this study was provided by the participants’ legal guardians/next of kin. Written informed consent was obtained from the individual(s) for the publication of any potentially identifiable images or data included in this article.

## Author contributions

TK: Writing – review & editing, Writing – original draft, Methodology, Investigation, Data curation, Conceptualization. TsI: Writing – review & editing, Writing – original draft, Supervision, Methodology, Investigation, Data curation, Conceptualization. TF: Writing – review & editing, Writing – original draft, Investigation. HiM: Writing – review & editing, Writing – original draft, Investigation. TG: Writing – review & editing, Writing – original draft, Investigation. CS: Writing – review & editing, Writing – original draft, Investigation. HaM: Conceptualization, Formal analysis, Funding acquisition, Methodology, Writing – original draft, Writing – review & editing. TeI: Conceptualization, Formal analysis, Funding acquisition, Investigation, Methodology, Supervision, Writing – original draft, Writing – review & editing. SK: Writing – review & editing, Methodology, Writing – original draft, Supervision, Conceptualization.

## References

[1] Ikezono T Shindo S Sekiguchi S Hanprasertpong C Li L Pawankar R. Cochlin-tomoprotein, a novel perilymph specific protein and a potential marker for the diagnosis of perilymphatic fistula. Audiol Neurootol. (2009) 14:338–44. doi: 10.1159/000212113, PMID: 19372652

[2] Ikezono T Matsumura T Matsuda H Shikaze S Saitoh S Shindo S. The diagnostic performance of a novel ELISA for human CTP (Cochlin-tomoprotein) to detect perilymph leakage. PLoS One. (2018) 13:e0191498. doi: 10.1371/journal.pone.0191498, PMID: 29377910 PMC5788340

[3] Kakehata S Sasaki A Futai K Kitani R Shinkawa H. Daily short-term intratympanic dexamethasone treatment alone as an initial or salvage treatment for idiopathic sudden sensorineural hearing loss. Audiol Neurootol. (2011) 16:191–7. doi: 10.1159/000320269, PMID: 20962524

[4] Matsuda H Sakamoto K Matsumura T Saito S Shindo S Fukushima K. A nationwide multicenter study of the Cochlin Tomo-protein detection test: clinical characteristics of perilymphatic fistula cases. Acta Otolaryngol. (2017) 137:S53–9. doi: 10.1080/00016489, PMID: 28368720

[5] Kanzaki J Inoue Y Ogawa K Fukuda S Fukushima K Gyo K. Effect of single-drug treatment on idiopathic sudden sensorineural hearing loss. Auris Nasus Larynx. (2003) 30:123–7. doi: 10.1016/S0385-8146(03)00009-9, PMID: 12753981

[6] Sasaki A Ikezono T Matsuda H Araki R Matsumura T Saitoh S. Prevalence of Perilymphatic fistula in patients with sudden-onset sensorineural hearing loss as diagnosed by Cochlin-Tomoprotein (CTP) biomarker detection: its association with age, hearing severity, and treatment. Eur Arch Otorrinolaringol. (2024) 257:490–2. PMID: 38123733 10.1007/s00405-023-08368-0 PMC11024054

[7] Tarabichi M. Endoscopic management of acquired cholesteatoma. Am J Otol. (1997) 18:544–9. PMID: 9303149

[8] Ito T Kubota T Watanabe T Futai K Furukawa T Kakehata S. Transcanal endoscopic ear surgery for pediatric population with a narrow external auditory canal. Int J Pediatr Otorhinolaryngol. (2015) 79:2265–9. doi: 10.1016/j.ijporl.2015.10.019, PMID: 26527072

[9] Morita Y Tono T Sakagami M Yamamoto Y Matsuda K Komori M. Nationwide survey of congenital cholesteatoma using staging and classification criteria for middle ear cholesteatoma proposed by the Japan Otological society. Auris Nasus Larynx. (2019) 46:346–52. doi: 10.1016/j.anl.2018.10.015, PMID: 30416024

[10] Matsuda H Tanzawa Y Sekine T Matsumura T Saito S Shindo S. Congenital membranous stapes footplate producing episodic pressure-induced Perilymphatic fistula symptoms. Front Neurol. (2020) 11:585747. doi: 10.3389/fneur.2020.585747, PMID: 33240208 PMC7683612

[11] Sarna B Abouzari M Merna C Jamshidi S Saber T Djalilian HR. Perilymphatic fistula: a review of classification, etiology, diagnosis, and treatment. Front Neurol. (2020) 11:11. doi: 10.3389/fneur.2020.01046, PMID: 33041986 PMC7522398

[12] Heilen S Lang CP Warnecke A Lenarz T Durisin M. Exploratory tympanotomy in sudden sensorineural hearing loss for the identification of a perilymphatic fistula – retrospective analysis and review of the literature. J Laryngol Otol. (2020) 134:501–8. doi: 10.1017/S0022215120000948 32614760

[13] Prenzler NK Schwab B Kaplan DM El-Saied S. The role of explorative tympanotomy in patients with sudden sensorineural hearing loss with and without perilymphatic fistula. Am J Otolaryngol. (2018) 39:46–9. doi: 10.1016/j.amjoto.2017.10.006, PMID: 29055686

[14] Matsuda H Hornibrook J Ikezono T. Assessing the efficacy of perilymphatic fistula repair surgery in alleviating vestibular symptoms and associated auditory impairments. Front Neurol. (2023) 14:14. doi: 10.3389/fneur.2023.1269298, PMID: 37900598 PMC10600483

